# DNA–Gold Nanozyme-Modified Paper Device for Enhanced Colorimetric Detection of Mercury Ions

**DOI:** 10.3390/bios10120211

**Published:** 2020-12-18

**Authors:** Min-Xin Mao, Rong Zheng, Chi-Fang Peng, Xin-Lin Wei

**Affiliations:** 1 State Key Laboratory of Dairy Biotechnology, Shanghai Engineering Research Center of Dairy Biotechnology, Dairy Research Institute, Bright Dairy & Food Co., Ltd., Shanghai 200436, China; 6190112080@jiangnan.edu.cn; 2 School of Food Science and Technology, Jiangnan University, Wuxi 214122, China; 6170112120@stu.jiangnan.edu.cn; 3 School of Agriculture and Biology, Shanghai Jiaotong University, Shanghai 200240, China; weixinlin@sjtu.edu.cn

**Keywords:** paper device, signal enhancement, mercury ion, colorimetric detection

## Abstract

In this work, a paper device consisted of a patterned paper chip, wicking pads, and a base was fabricated. On the paper chip, DNA–gold nanoparticles (DNA–AuNPs) were deposited and Hg^2+^ ions could be adsorbed by the DNA–AuNPs. The formed DNA–AuNP/Hg^2+^ nanozyme could catalyze the tetramethylbenzidine (TMB)–H_2_O_2_ chromogenic reaction. Due to the wicking pads, a larger volume of Hg^2+^ sample could be applied to the paper device for Hg^2+^ detection and therefore the color response could be enhanced. The paper device achieved a cut-off value of 50 nM by the naked eye for Hg^2+^ under optimized conditions. Moreover, quantitative measurements could be implemented by using a desktop scanner and extracting grayscale values. A linear range of 50–2000 nM Hg^2+^ was obtained with a detection limit of 10 nM. In addition, the paper device could be applied in the detection of environmental water samples with high recoveries ranging from 85.7% to 105.6%. The paper-device-based colorimetric detection was low-cost, simple, and demonstrated high potential in real-sample applications.

## 1. Introduction

Mercury ions (Hg^2+^) are one of toxic heavy metals. They are widely found in the environment [1], are a serious threat to human health [2]. In order to control the risk of Hg^2+^, the US Environmental Protection Agency (EPA) and the World Health Organization (WHO) set a maximum contents of Hg^2+^ in drinking water which are 2.0 μg/L (10 nM) and 6.0 μg/L (30 nM), respectively [3]. In practice, numerous conventional lab-dependent techniques such as inductively-coupled plasma mass spectrometry (ICP-MS) [4], atomic fluorescence spectrometry (AFS) [5], and high performance liquid chromatography (HPLC) [6,7] have been well-established for detection of Hg^2+^. However, their operations are highly dependent on time-consuming sample pretreatments, expensive instrumentation, and skilled technicians, making them unsuitable for rapid and on-site detection of target Hg^2+^ ions [8,9].

In recent years, many researchers have established a large number of methods for the detection of Hg^2+^ based on nanomaterials, such as fluorescent [10], colorimetric [11], chemiluminescent [12], surface-enhanced raman spectroscopy (SERS), and electrochemical methods [13,14]. The above methods have demonstrated many advantages, such as rapidness and high sensitivity. Hg^2+^ can strongly interact with many nanomaterials such as gold nanoparticles (AuNPs), gold nanorods, and silver nanoparticles. When these nanoparticles adsorb Hg^2+^, Au–Hg nano-alloys or Ag–Hg nano-alloys could be formed [15]. Some researchers have reported that Au–Hg nano-alloys possess peroxidase-like property and could catalyze H_2_O_2_-mediated oxidation of tetramethylbenzidine (TMB) [16]. We reported that the DNA–AuNP complex could capture Hg^2+^ and form DNA–Au–Hg nano-alloys [17]. These DNA–Au–Hg nano-alloys demonstrated much stable peroxidase-like activity and could achieve highly-sensitive colorimetric detection of Hg^2+^.

Since Whiteside’s group first proposed a paper-based device for the detection of biochemicals in blood, this device has received considerable attention by many researchers [18,19]. Paper is an excellent substrate material for sample filtration and preconcentration [20,21] due to its high surface-to-volume ratio, low-cost, and portability [22,23]. It has also been widely used in medical diagnosis [24,25], environmental monitoring [26], and food quality analysis [27], etc. Paper can be modified by various nanomaterials, such as ceria nanoparticles [28], AuNPs [29], silver nanoparticles [30,31], and carbon nanotubes [32,33,34] in order to develop assays for various targets or improve colorimetric homogeneity and intensity. For example, He et al. successfully developed an ultrasensitive nucleic acid biosensor based on HRP–AuNP dual labels and a lateral flow strip biosensor [35]. Qiao et al. developed a fluorometric Hg^2+^ test strip using Au–Ag nanoclusters as fluorescent probes combined with suppressing “coffee stains” by a bio-inspired fabrication strategy [36]. Zhang et al. used Cy5-labeled functional ssDNA toward multiple analytes, graphene oxide, and paper substrate to fabricate a paper device to report the presence of the Hg^2+^ and Ag^+^ ions and aminoglycoside antibiotics in food [37]. Li et al. prepared three kinds of doped carbon quantum dots and fabricated a smartphone-based three-channel ratio fluorescence device for simultaneous determination of Hg^2+^, Fe^3+^, and Cu^2+^ ions in environmental samples [38]. Zhou et al. developed a rapid and sensitive paper-based analytical device (PAD) to detect the total tetracyclines in environmental water based on a paper channel by field amplification stacking and fluorescent imaging [39].

In this paper, DNA–AuNPs were deposited onto filter paper and a nanozyme-based colorimetric detection of Hg^2+^ was carefully optimized on the filter paper. The detection was eventually carried out on a paper chip, which had detection zones modified with DNA–AuNPs and connected to a substrate reservoir by multiple channels [40]. Layers of filter paper as a wicking pad were placed under the detection zones to facilitate Hg^2+^ enrichment. This paper device demonstrated advantages including being simple, low-cost, and sensitive.

## 2. Experimental

### 2.1. Reagents and Instruments

Chloroauric acid (HAuCl_4_) and sodium citrate were purchased from Sigma-Aldrich (Shanghai, China) and 3,3′,5,5′-tetramethylbenzidine (TMB) and hydrogen peroxide were purchased from Aladdin Reagent Company (Shanghai, China). All metal ion standard solutions were purchased from the National Institute of Metrology P. R. China. All other reagents were of analytical grade. Whatman No. 1 filter paper was obtained from GE Healthcare (Shanghai, China). Ultra-pure water was prepared with a Milli-Q pure system for all the experiments.

UV–visible (UV–vis) absorption spectra were measured with an Agilent Cary 60 UV–vis spectrophotometer (Crawford Scientific, Strathaven, UK) at room temperature. Absorption values of reaction solutions were obtained with a microplate reader (Bio-Tek, Elx800, Winooski, VT, USA). Transmission electron microscopy (TEM) images were obtained on a JEOL JEM-2100 at an accelerating voltage of 200 kV.

### 2.2. Fabrication of Gold Nanozyme Paper Device

**Figure 1:**
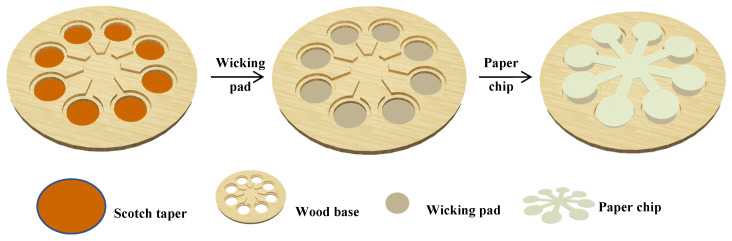
Schematic of paper device fabrication.

The pattern of paper chip was designed using CorelDRAW software. As shown in Figure S1a, the paper chip had a substrate reservoir, which connected with eight detection zones through eight channels. The pattern of the paper device base was similar to the pattern of the paper chip, except for having eight smaller holes located at the center of each detection zone (Figure 1).

The filter paper was cut into the paper chip according to the designed pattern by a CO_2_ laser engraving machine (Golden, CO 80403 USA). Then, the obtained paper cuttings were immersed into ultrapure water, rinsed for 30 s, and then dried at 40 °C for later use.

A wood board was engraved by the CO_2_ laser to produce the pattern (Figure S1b). The depth of the groove for paper chip was set at 1.5 mm and other sizes are shown in the pattern. The engraved wood board was immersed in 1% paraffin solution (dissolved in n-hexane) for 5 min, and then baked at 80 °C for 10 min. The fabrication of the paper device is shown in Figure 1. Firstly, Scotch tape was attached to back side of the pretreated wood base. Layers of round filter paper as a wicking pad were filled into the holes of the wood board. Then the paper chip was fixed closely to the patterned wood board. The images of the paper device are shown in Figure S2.

### 2.3. Colorimetric Detection of Hg^2+^ on Paper Device

The DNA–AuNP complex were prepared according to our previous reports [17,41]. To each detection zone on paper chip, 2 μL of the DNA–AuNPs (0.6 nM) was added. After being dried at room temperature for 5 min, 20–100 μL of standard Hg^2+^ solution or sample was added to each sample detection zone. After being incubated for 20 min, 300 μL of substrate (0.4 mM TMB and 3.0% H_2_O_2_ in 0.1 M citric buffer) was added to the reagent reservoir. After the substrates were distributed to each detection zone, chromogenic reaction was initiated and continued for 20 min. The color development was recorded by mobile phone and desktop scanning, and the colorimetric signal was analyzed using Image J software.

### 2.4. Validation of the Colorimetric Detection

Tap water and lake water samples from Li Lake (Wuxi, China) were spiked with different concentrations (200, 500, and 1000 nM) of Hg^2+^, filtered twice through 0.22 µm membrane, and then measured by the paper device. The lake water samples were filtered with filter paper modified with graphene oxide, and then filtered with a 0.22 µm membrane to carry out the next detection.

## 3. Results and Discussion

### 3.1. Fabrication of Paper Device

Generally, the sensitivity of paper-based assays is negatively affected by small volumes of sample loaded onto a small-size detection zone [20,35,42]. The volume of loaded sample could be increased significantly through adopting water adsorbent, thereby improving the detection sensitivity [22]. In order to achieve enhanced sensitivity in our designed paper chip for Hg^2+^ colorimetric detection, we used a base to hold the paper chip and wicking pad. The patterns of paper chip and wood base were both easily produced. The cost of one paper chip is about 15 cents (CNY). The cost of the wood base is about twenty cents and it could be reusable. Thus, the device is low-cost. The wood base and paper chip were obtained through laser engraving as shown in Figure 1. The size of the paper chip and wood base could be easily controlled to match with each other. On the wood base, it was much simpler to cut a hole than engrave a well. In order to fix the wicking pad, scotch tape was used to seal the holes.

**Figure 2:**
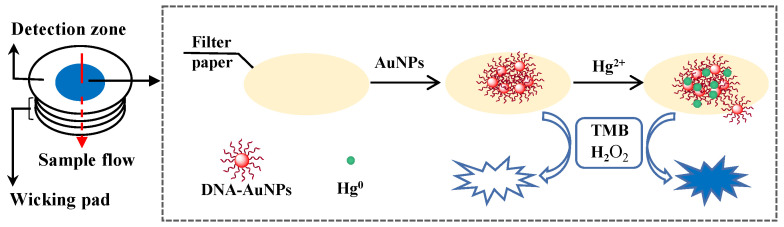
Schematic diagram of Hg^2+^ detection.

In order to prevent rapid sample leakage along the wood base surface, the inner surface of the base was hydrophobically modified by coating with paraffin. As shown in Figure S3, the contact angle to the waterdrop on the wood board surface was over 90 degree and the water drop on the base surface could be kept stable for over 60 min. These results confirmed the good hydrophobicity of the paraffin-modified base, which facilitated stable sample flow vertical from paper chip to the wicking pad and Hg^2+^ absorption by the DNA–AuNPs on the detection zone (Figure 2).

### 3.2. Colorimetric Detection of Hg^2+^

The DNA–AuNPs had peroxidase-like activity and could catalyze the chromogenic reaction of TMB–H_2_O_2_, but the catalytic activity was weak. The peroxidase activity of the DNA–AuNPs could be significantly enhanced after the DNA–AuNPs adsorbed Hg^2+^ [15] and produced a very strong peak of TMB–H_2_O_2_ at 650 nm (Figure S4).

**Figure 3:**
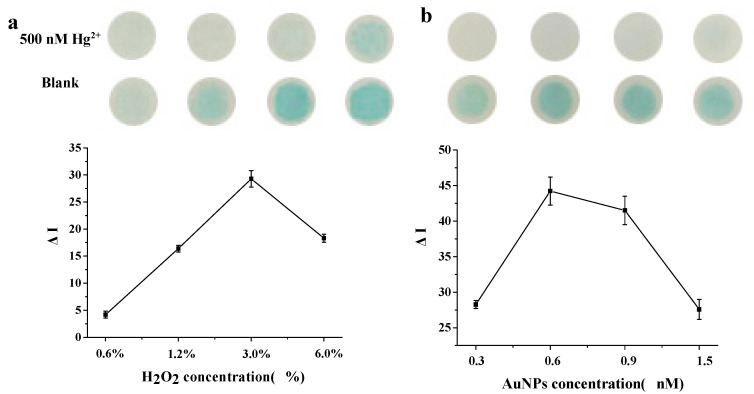
Optimization of H_2_O_2_ and DNA–AuNP concentration. (**a**) Effect of H_2_O_2_ concentration and (**b**) effect of DNA–AuNP concentration.

In order to obtain sensitive detection on the paper chip, the effect of H_2_O_2_ concentration and DNA–AuNP concentration were investigated. The optimal conditions were evaluated by the colorimetric intensity difference, ∆I = I − I_0_ (I and I_0_ refer to the gray value obtained with and without Hg^2+^). As shown in Figure 3a,b, the highest color intensity could be obtained with 3% H_2_O_2_ and 0.6nM DNA–AuNPs, respectively.

**Figure 4:**
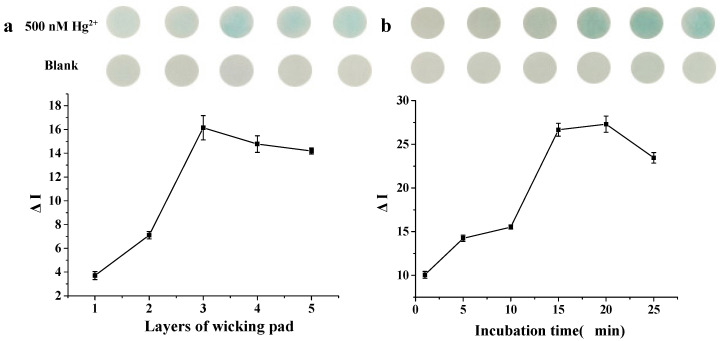
Optimization of the Hg^2+^ volume and adsorption time. (**a**) Effect of layers of wicking pad and (**b**) effect of incubation time.

The Hg^2+^ volume was also investigated. When more than 20 μL was applied onto the detection zone, Hg^2+^ solution would overflow to the substrate reservoir, resulting in uncontrolled color development. With the superimposed wicking pad under paper chip, the volume of Hg^2+^ solution could be increased linearly with the increasing layers of wicking pad. In order to simplify the operation, five layers of wicking pad and 100 μL of Hg^2+^ solution at most were investigated. As shown in Figure 4a, it was found that darkest blue appeared when 60 μL of Hg^2+^ solution was used. Compared with 20 μL of Hg^2+^ solution, 60 μL was suitable for paper chip alone, and the ∆I increased four-fold. Unfortunately, no higher signal increasement was found when over 60 μL of Hg^2+^ solution was dropped onto the paper chip. These results were probably due to the fact that part of the DNA–AuNPs could be washed away by excessive Hg^2+^ solution. After incubating with Hg^2+^ for 15–20 min, the highest colorimetric intensity could be obtained when 60 μL of Hg^2+^ solution was used (Figure 4b).

**Figure 5:**
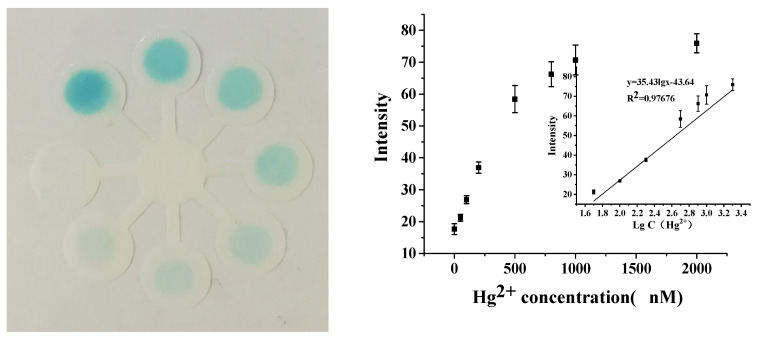
The image of detection of Hg^2+^ and calibration curve of colorimetric detection.

As shown in Figure 5, the color intensity increased with the increased Hg^2+^ concentration on the paper device, and 50 nM Hg^2+^ could be distinguished by the naked eye. With the desktop scanning, quantitative determination could be implemented. A linear relationship between the gray intensity and logarithm of Hg^2+^ concentration could be obtained in the range of 0.05–2 μM. A detection limit of 10 nM was achieved, based on a 3σ/slope, where σ was the standard deviation of blank samples. Compared with some typical nanomaterial-modified papers or test strips for Hg^2+^ colorimetric detection, the above paper-device-based detection demonstrated comparable sensitivity (Table S1).

**Figure 6:**
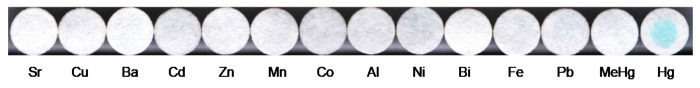
Selectivity of the method toward heavy metal ions.

To explore the selectivity of this colorimetric detection, various common metal ions including MeHg^+^, Mn^2+^, Cu^2+^, Ni^2+^, Ba^2+^, Cd^2+^, Al^3+^, Zn^2+^, Fe^3+^, Cr^3+^, Co^2+^, Sr^2+^, and Bi^3+^ were tested. As shown in Figure 6, Hg^2+^ ions (1 μM) showed a deep blue color in the paper and negligible color responses were observed toward the other metal ions (10 μM), indicating that the high selectivity of this method was toward Hg^2+^.

### 3.3. Application in Real Samples

To verify the feasibility of this paper device in detecting real samples, tap water and lake water samples were spiked with Hg^2+^ and applied to the paper device. The results were obtained as shown in Table 1. The recoveries ranged from 85.7% to 105.6% when water samples spiked with 200, 500, and 1000 nM Hg^2+^ were measured. These results showed the great potential of this paper device for Hg^2+^ detection in practical applications.

## 4. Conclusions

In conclusion, a paper device consisting of a patterned paper chip and a base were successfully fabricated. The designed paper chip and wicking pad on the paper device facilitated the operation of DNA–gold nanozyme-based colorimetric detection of Hg^2+^ and enhanced the sensitivity. The color development of 50 nM Hg^2+^on the paper device could be distinguished by the naked eye. Moreover, quantitative analysis of the color could be implemented by desktop scanner and gray intensity extracting. The colorimetric detection of Hg^2+^ was a low-cost, simple operation that demonstrated great potential in real sample detection. In addition, the paper device could be extended to combine with other nanosensors for more applications.

## Figures and Tables

**Table 1:** Determination of Hg^2+^ in tap water and lake water samples. (n = 3).

Sample	Added (nM)	Detected (nM)	Recovery (%)	RSD (%)
Tap water	200	189.8	94.9	3.6
	500	506.8	101.4	2.9
	1000	856.9	85.7	2.0
Lake water	200	197.6	98.8	4.7
	500	483.7	96.7	4.2
	1000	1056.0	105.60	2.7

## Supplementary Materials

The following are available online at https://www.mdpi.com/2079-6374/10/12/211/s1, Figure S1: Designed pattern of (**a**) paper chip and (**b**) base of the paper device; Figure S2: Photographs of paper-based device. (**a**) paper chip; (**b**) wood base; (**c**) wood base filled with wicking pad and (**d**) paper device; Figure S3: Photograph of a water drop on wood board surface; Figure S4: UV- vis absorption spectra of TMB-H_2_O_2_ reaction. (**a**) DNA-AuNPs without Hg^2+^; (**b**) DNA-AuNPs with Hg^2+^. Table S1: Comparison of paper-based devices for the detection of Hg^2+^ reported in literatures.

## References

[1] Li X., Zhang Y., Chang Y., Xue B., Kong X., Chen W. (2017). Catalysis-reduction strategy for sensing inorganic and organic mercury based on gold nanoparticles. Biosens. Bioelectron..

[2] Deng L., Li Y., Yan X., Xiao J., Ma C., Zheng J., Liu S., Yang R. (2015). Ultrasensitive and Highly Selective Detection of Bioaccumulation of Methyl-Mercury in Fish Samples via Ag-0/Hg-0 Amalgamation. Anal. Chem..

[3] Kan C., Shao X., Song F., Xu J., Zhu J., Du L. (2019). Bioimaging of a fluorescence rhodamine-based probe for reversible detection of Hg (II) and its application in real water environment. Microchem. J..

[4] Rofouei M.K., Rezaei A., Masteri-Farahani M., Khani H. (2012). Selective extraction and preconcentration of ultra-trace level of mercury ions in water and fish samples using Fe3O4-magnetite-nanoparticles functionalized by triazene compound prior to its determination by inductively coupled plasma-optical emission spectrometry. Anal. Methods.

[5] Carneado S., Pero-Gascon R., Ibanez-Palomino C., Lopez-Sanchez J.F., Sahuquillo A. (2015). Mercury(II) and methylmercury determination in water by liquid chromatography hyphenated to cold vapour atomic fluorescence spectrometry after online short-column preconcentration. Anal. Methods.

[6] Zhou Q., Xing A., Zhao K. (2014). Simultaneous determination of nickel, cobalt and mercury ions in water samples by solid phase extraction using multiwalled carbon nanotubes as adsorbent after chelating with sodium diethyldithiocarbamate prior to high performance liquid chromatography. J. Chromatogr. A.

[7] Wang L., Zhou J.-B., Wang X., Wang Z.-H., Zhao R.-S. (2016). Simultaneous determination of copper, cobalt, and mercury ions in water samples by solid-phase extraction using carbon nanotube sponges as adsorbent after chelating with sodium diethyldithiocarbamate prior to high performance liquid chromatography. Anal. Bioanal. Chem..

[8] Anand T., Sankar M. (2020). A dual colorimetric chemosensor for Hg(II) and cyanide ions in aqueous media based on a nitrobenzoxadiazole (NBD)-antipyrine conjugate with INHIBIT logic gate behaviour. Anal. Methods.

[9] Feng X., Zhang J., Wang J., Han A., Fang G., Liu J., Wang S. (2020). The stabilization of fluorescent copper nanoclusters by dialdehyde cellulose and their use in mercury ion sensing. Anal. Methods.

[10] Chen L., Fu X., Lu W., Chen L. (2013). Highly Sensitive and Selective Colorimetric Sensing of Hg^2+^ Based on the Morphology Transition of Silver Nanoprisms. ACS Appl. Mater. Interfaces.

[11] Long Y.J., Li Y.F., Liu Y., Zheng J.J., Tang J., Huang C.Z. (2011). Visual observation of the mercury-stimulated peroxidase mimetic activity of gold nanoparticles. Chem. Commun..

[12] Cai S., Lao K., Lau C., Lu J. (2011). “Turn-On” Chemiluminescence Sensor for the Highly Selective and Ultrasensitive Detection of Hg^2+^ Ions Based on Interstrand Cooperative Coordination and Catalytic Formation of Gold Nanoparticles. Anal. Chem..

[13] Song C., Yang B., Yu Z., Yang Y., Wang L. (2017). Ultrasensitive sliver nanorods array SERS sensor for mercury ions. Biosens. Bioelectron..

[14] He Z.-J., Kang T.-F., Lu L.-P., Cheng S.-Y. (2020). An electrochemiluminescence sensor based on CdSe@CdS-functionalized MoS2 and a GOD-labeled DNA probe for the sensitive detection of Hg(ii). Anal. Methods.

[15] Long F., Zhu A., Shi H. (2013). Recent Advances in Optical Biosensors for Environmental Monitoring and Early Warning. Sensors.

[16] Tan L., Zhang Y., Qiang H., Li Y., Sun J., Hu L., Chen Z. (2016). A sensitive Hg(II) colorimetric sensor based on synergistic catalytic effect of gold nanoparticles and Hg. Sens. Actuator B Chem..

[17] Peng C.-F., Pan N., Xie Z.-J., Wu L.-L. (2016). Highly sensitive and selective colorimetric detection of Hg^2+^ based on the separation of Hg^2+^ and formation of catalytic DNA–gold nanoparticles. Anal. Methods.

[18] Martinez A.W., Phillips S.T., Whitesides G.M. (2008). Three-dimensional microfluidic devices fabricated in layered paper and tape. Proc. Natl. Acad. Sci. USA.

[19] Fu E., Downs C. (2017). Progress in the development and integration of fluid flow control tools in paper microfluidics. Lab Chip.

[20] Nilghaz A., Lu X. (2019). Detection of antibiotic residues in pork using paper-based microfluidic device coupled with filtration and concentration. Anal. Chim. Acta.

[21] Pena-Pereira F., Lavilla I., Bendicho C. (2016). Paper-based analytical device for instrumental-free detection of thiocyanate in saliva as a biomarker of tobacco smoke exposure. Talanta.

[22] Feng L., Li X., Li H., Yang W., Chen L., Guan Y. (2013). Enhancement of sensitivity of paper-based sensor array for the identification of heavy-metal ions. Anal. Chim. Acta.

[23] Evans E., Moreira Gabriel E.F., Benavidez T.E., Tomazelli Coltro W.K., Garcia C.D. (2014). Modification of microfluidic paper-based devices with silica nanoparticles. Analyst.

[24] Jeong S.-G., Lee S.-H., Choi C.-H., Kim J., Lee C.-S. (2015). Toward instrument-free digital measurements: A three-dimensional microfluidic device fabricated in a single sheet of paper by double-sided printing and lamination. Lab Chip.

[25] Yetisen A.K., Akram M.S., Lowe C.R. (2013). Paper-based microfluidic point-of-care diagnostic devices. Lab Chip.

[26] Liu H., Crooks R.M. (2011). Three-Dimensional Paper Microfluidic Devices Assembled Using the Principles of Origami. J. Am. Chem. Soc..

[27] Ishii S., Segawa T., Okabe S. (2013). Simultaneous Quantification of Multiple Food- and Waterborne Pathogens by Use of Microfluidic Quantitative PCR. Appl. Environ. Microbiol..

[28] Ornatska M., Sharpe E., Andreescu D., Andreescu S. (2011). Paper Bioassay Based on Ceria Nanoparticles as Colorimetric Probes. Anal. Chem..

[29] Lee Y.-F., Huang C.-C. (2011). Colorimetric Assay of Lead Ions in Biological Samples Using a Nanogold-Based Membrane. ACS Appl. Mater. Interfaces.

[30] Ratnarathorn N., Chailapakul O., Henry C.S., Dungchai W. (2012). Simple silver nanoparticle colorimetric sensing for copper by paper-based devices. Talanta.

[31] Chaiyo S., Siangproh W., Apilux A., Chailapakul O. (2015). Highly selective and sensitive paper-based colorimetric sensor using thiosulfate catalytic etching of silver nanoplates for trace determination of copper ions. Anal. Chim. Acta.

[32] Esmaeili N., Rakhtshah J., Kolvari E., Shirkhanloo H. (2020). Ultrasound assisted-dispersive-modification solid-phase extraction using task-specific ionic liquid immobilized on multiwall carbon nanotubes for speciation and determination mercury in water samples. Microchem. J..

[33] Figueredo F., Garcia P.T., Corton E., Coltro W.K.T. (2016). Enhanced Analytical Performance of Paper Microfluidic Devices by Using Fe3O4 Nanoparticles, MWCNT, and Graphene Oxide. ACS Appl. Mater. Interfaces.

[34] Wang P., Ge L., Yan M., Song X., Ge S., Yu J. (2012). Paper-based three-dimensional electrochemical immunodevice based on multi-walled carbon nanotubes functionalized paper for sensitive point-of-care testing. Biosens. Bioelectron..

[35] He Y., Zhang S., Zhang X., Baloda M., Gurung A.S., Xu H., Zhang X., Liu G. (2011). Ultrasensitive nucleic acid biosensor based on enzyme-gold nanoparticle dual label and lateral flow strip biosensor. Biosens. Bioelectron..

[36] Qiao Y., Shang J., Li S., Feng L., Jiang Y., Duan Z., Lv X., Zhang C., Yao T., Dong Z. (2016). Fluorimetric Mercury Test Strips with Suppressed "Coffee Stains" by a Bio-inspired Fabrication Strategy. Sci. Rep..

[37] Zhang Y., Zuo P., Ye B.C. (2015). A low-cost and simple paper-based microfluidic device for simultaneous multiplex determination of different types of chemical contaminants in food. Biosens. Bioelectron..

[38] Li D., Sun Y., Shen Q., Zhang Q., Huang W., Kang Q., Shen D. (2020). Smartphone-based three-channel ratiometric fluorescent device and application in filed analysis of Hg^2+^, Fe^3+^ and Cu^2+^ in water samples. Microchem. J..

[39] Zhou T., Liu J.-J., Xu Y., Wu Z.-Y. (2019). Fast and sensitive screening detection of tetracyclines with a paper-based analytical device. Microchem. J..

[40] Han K.N., Choi J.S., Kwon J. (2017). Gold nanozyme-based paper chip for colorimetric detection of mercury ions. Sci. Rep..

[41] Xie Z.-J., Bao X.-Y., Peng C.-F. (2018). Highly Sensitive and Selective Colorimetric Detection of Methylmercury Based on DNA Functionalized Gold Nanoparticles. Sensors.

[42] Mei Z., Chu H., Chen W., Xue F., Liu J., Xu H., Zhang R., Zheng L. (2013). Ultrasensitive one-step rapid visual detection of bisphenol A in water samples by label-free aptasensor. Biosens. Bioelectron..

