# Supplementary material for: DNA–Gold Nanozyme-Modified Paper Device for Enhanced Colorimetric Detection of Mercury Ions
Source: Biosensors (Basel). 2020 Dec 18;10(12):211. doi: 10.3390/bios10120211 (PMC7766257; doi:10.3390/bios10120211)
Supplement: Supplementary file 1 [file biosensors-10-00211-s001.pdf]

DNA-gold Nanozyme Modified Paper Device for Enhanced Colorimetric Detection of Mercury Ions

Supplementary Material

Preparation of AuNPs

The AuNPs were prepared by sodium citrate reduction method[1], as follows: the conical flask of the composite material was soaked in aqua regia for 24 h and then washed with ultrapure water. Briefly, in a conical flask, 2.5 mL 4g/L $HAuCl_4$ was added into 97.5 mL ultrapure water, and heated to boiling while stirring. Then 2 mL of sodium citrate solution (1.0 %) was quickly added into the above mixture solution with vigorous stirring. After the burgundy was observed, the solution continued to be heated for 10 min, and then cooled down at room temperature, and stored at 4 ºC for later use.

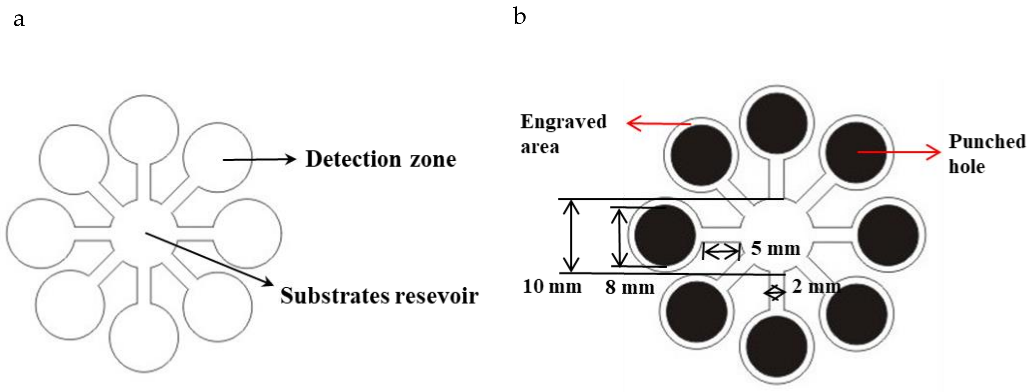

Fig. S1. Designed pattern of (**a**) paper chip and (**b**) base of the paper device.

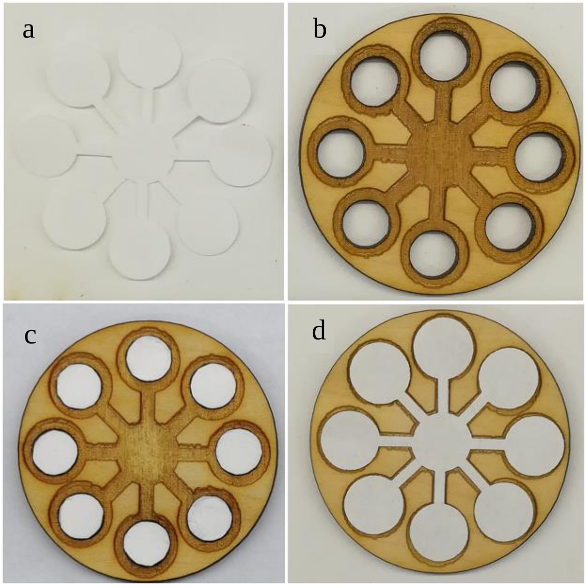

Fig. S2. Photographs of paper-based device. (**a**) paper chip; (**b**) wood base; (**c**) wood base filled with wicking pad and (**d**) paper device.

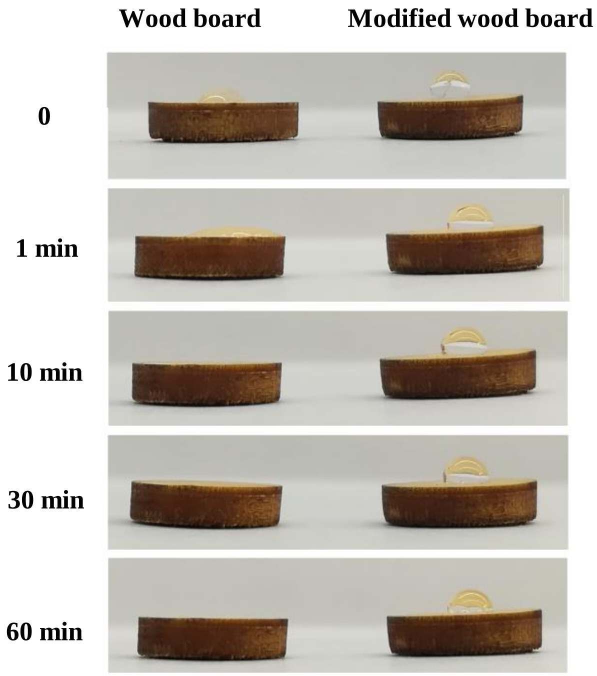

Fig. S3. Photograph of a water drop on wood board surface

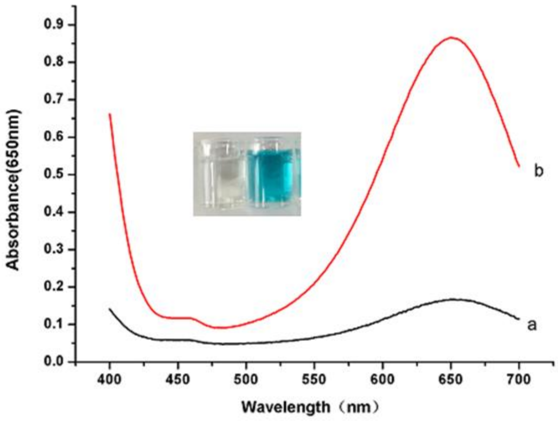

Figure 4. UV- vis absorption spectra of TMB-H_2O_2 reaction. (**a**) DNA-AuNPs without Hg^{2+}; (**b**) DNA-AuNPs with Hg^{2+}

Table S1 Comparison of paper-based devices for the detection of Hg^{2+} reported in literatures

Method	Reaction substrate	Detection probe	LOD of naked eye （nM）	Application	Reference
colorimetry	filter paper	AgNPs	600	drinking water	[2]
colorimetry	NC membrane	AuNPs-antibody	8.0	-	[3]
Fluorescence	filter paper	Rhodamine 6G	4980	-	[4]
colorimetry	NC membrane	AuNPs-DNA	6.0	tap water	[5]
colorimetry	filter paper	AuNPs	500	tap water	[6]
colorimetry	filter paper	AuNPs	50	tap water、lake water	Our work

References

1. Peng, C.F.; Pan, N.; Xie, Z.J.; Wu, L.L. Highly sensitive and selective colorimetric detection of Hg2+ based on the separation of Hg2+ and formation of catalytic DNA–gold nanoparticles. *Anal. Methods* **2016**, *8*, 1021-1025.
2. Apilux, A.; Siangproh, W.; Praphairaksit, N.; Chailapakul, O. Simple and rapid colorimetric detection of Hg(II) by a paper-based device using silver nanoplates. *Talanta* **2012**, *97*, 388-394.
3. Zhou, Y.; Li, Y.-S.; Meng, X.-Y.; Zhang, Y.-Y.; Yang, L.; Zhang, J.-H.; Wang, X.-R.; Lu, S.-Y.; Ren, H.-L.; Liu, Z.-S. Development of an immunochromatographic strip and its application in the simultaneous determination of Hg(II), Cd(II) and Pb(II). *Sens. Actuator B-Chem.* **2013**, *183*, 303-309.
4. Patidar, R.; Rebary, B.; Paul, P. Colorimetric and Fluorogenic Recognition of Hg2+ and Cr3+ in Acetonitrile and their Test Paper Recognition in Aqueous Media with the Aid of Rhodamine Based Sensors. *J. Fluoresc.* **2015**, *25*, 387-395.
5. Yang, F.; Duan, J.; Li, M.; Wang, Z.; Guo, Z. Visual and On-site Detection of Mercury(II) Ions on Lateral Flow Strips Using DNA-functionalized Gold Nanoparticles. *Anal. Sci.* **2012**, *28*, 333-338.
6. Han, K.N.; Choi, J.-S.; Kwon, J. Gold nanozyme-based paper chip for colorimetric detection of mercury ions. *Sci Rep* **2017**, *7*.
